# Synthetically Lethal Interactions of Heme Oxygenase-1 and Fumarate Hydratase Genes

**DOI:** 10.3390/biom10010143

**Published:** 2020-01-16

**Authors:** Paulina Podkalicka, Olga Mucha, Szczepan Kruczek, Anna Biela, Kalina Andrysiak, Jacek Stępniewski, Maciej Mikulski, Michał Gałęzowski, Kamil Sitarz, Krzysztof Brzózka, Alicja Józkowicz, Józef Dulak, Agnieszka Łoboda

**Affiliations:** 1 Department of Medical Biotechnology, Faculty of Biochemistry, Biophysics and Biotechnology, Jagiellonian University, Gronostajowa 7, 30-387 Kraków, Poland; paulina.podkalicka@doctoral.uj.edu.pl (P.P.); olga.mucha@doctoral.uj.edu.pl (O.M.); kruczek.szczepan@gmail.com (S.K.); anna.biela@uj.edu.pl (A.B.); kalina.andrysiak@doctoral.uj.edu.pl (K.A.); jacek.stepniewski@uj.edu.pl (J.S.); alicja.jozkowicz@uj.edu.pl (A.J.); jozef.dulak@uj.edu.pl (J.D.); 2 Ryvu Therapeutics S.A., Bobrzyńskiego 14, 30-348 Kraków, Poland; maciej.mikulski@ryvu.com (M.M.); michal.galezowski@ryvu.com (M.G.); kamil.sitarz@ryvu.com (K.S.); krzysztof.brzozka@ryvu.com (K.B.)

**Keywords:** heme oxygenase-1, fumarate hydratase, small-molecule inhibitors, synthetic lethality hereditary leiomyomatosis and renal cell carcinoma

## Abstract

Elevated expression of heme oxygenase-1 (HO-1, encoded by *HMOX1*) is observed in various types of tumors. Hence, it is suggested that HO-1 may serve as a potential target in anticancer therapies. A novel approach to inhibit HO-1 is related to the synthetic lethality of this enzyme and fumarate hydratase (FH). In the current study, we aimed to validate the effect of genetic and pharmacological inhibition of HO-1 in cells isolated from patients suffering from hereditary leiomyomatosis and renal cell carcinoma (HLRCC)—an inherited cancer syndrome, caused by FH deficiency. Initially, we confirmed that UOK 262, UOK 268, and NCCFH1 cell lines are characterized by non-active FH enzyme, high expression of Nrf2 transcription factor-regulated genes, including *HMOX1* and attenuated oxidative phosphorylation. Later, we demonstrated that shRNA-mediated genetic inhibition of *HMOX1* resulted in diminished viability and proliferation of cancer cells. Chemical inhibition of HO activity using commercially available inhibitors, zinc and tin metalloporphyrins as well as recently described new imidazole-based compounds, especially SLV-11199, led to decreased cancer cell viability and clonogenic potential. In conclusion, the current study points out the possible relevance of HO-1 inhibition as a potential anti-cancer treatment in HLRCC. However, further studies revealing the molecular mechanisms are still needed.

## 1. Introduction

Hereditary leiomyomatosis and renal cell cancer (HLRCC) is a cancer syndrome, caused by inherited, autosomal dominant mutation in the fumarate hydratase (*FH*) gene, described for the first time in 2002 by Tomlinson et al. [1]. Loss-of-function mutation in the tumor-suppressor *FH* gene might lead to cutaneous leiomyomas, uterine fibroids, and aggressive kidney cancers with early onset of metastasis [2]. Despite growing interest and effort put on investigations on HLRCC disease, no standard therapy is currently available [3]. FH is an enzyme involved in the tricarboxylic acid cycle (TCA) in which it catalyzes the reversible conversion of fumarate into malate. At the molecular level, FH-deficiency causes perturbations in the mitochondrial Krebs cycle leading to decreased oxidative phosphorylation and enhanced aerobic glycolysis as an adaptation for maintaining sufficient energy production, known as the Warburg effect. Such a metabolic switch is independent of the oxygen level [4] and leads to the accumulation of fumarate which has been implicated in numerous metabolic alterations. Despite the fact that various oncogenic pathways have been suggested to be involved, the primary mechanism responsible for HLRCC-associated renal tumor development remains elusive. It was shown that fumarate, acting as a competitive inhibitor of the prolyl hydroxylases (PHDs), stabilizes hypoxia-inducible factor-1α (HIF-1α) at normal oxygen tension. This leads to the upregulation of hypoxia-related genes, such as vascular endothelial growth factor (VEGF) and glucose-transporter-1 (GLUT1) which accelerate the aggressive phenotype of HLRCC-related kidney tumors [5]. Noteworthy, glycolytic shift and increased intracellular glucose level promote reactive oxygen species (ROS) formation, which further contributes to the stabilization of HIFs [6]. Moreover, elevated intracellular ROS production was suggested to sensitize HLRCC-related cancer cells to pro-oxidant anti-cancer therapies, such as bortezomib treatment [7].

Although the hypoxia pathway accelerates the aggressiveness of HLRCC tumors, the opinion that this mechanism is a primary cause of HLRCC-related kidney cancer development is rather controversial [8]. Comprehensive in vivo study with animals having conditionally inactivated *HIF-1α*, *HIF-2α*, and *FH* genes revealed renal cyst formation and tumor development as being rather HIF-independent. Instead, other potential pathways have been highlighted. In 2016, it was shown that elevated levels of fumarate in the cells cause epigenetic suppression, which leads to epithelial-to-mesenchymal transition and promotes tumor metastasis [9]. Moreover, fumarate accumulation with a more acidic environment (being the result of the glycolytic switch) was suggested to enhance the succination process in HLRCC [10]. Succination is an irreversible, post-translation modification, which involves the reaction of fumarate with a cysteine group of proteins. Indeed, analysis of two FH-deficient cell lines—UOK 262 and NCCFH1—as well as FH-deficient tumor, showed strong succination of various, functionally important proteins, including glyceraldehyde 3-phosphate dehydrogenase (GAPDH) and Kelch-like ECH-associated protein 1 (Keap1) [11]. The consequence of the latter, namely the release of the Nrf2 transcription factor from Keap1, its translocation to the nucleus, and activation of anti-oxidant genes, is thought to play an important, if not causal role in HLRCC-related kidney cancer development [12]. Heme oxygenase-1 (HO-1, encoded by *HMOX1* gene), which is one of the transcription targets of Nrf2, was shown to be significantly upregulated both in FH-deficient cells and in a mouse model of FH-deficiency [13].

HO-1 and the products of its activity, carbon monoxide (CO), ferrous ions (Fe^2+^), and biliverdin (quickly reduced to bilirubin) exert numerous cytoprotective activities including anti-oxidant, anti-apoptotic, anti-inflammatory, and pro-angiogenic effects. It has been shown that all those beneficial features of HO-1 are indispensable for not only normal but also for tumor cells. The regulatory role of HO-1 in tumor cell proliferation, survival, and metastasis has been confirmed in numerous types of cancer (reviewed in [14,15]). Interestingly, Frezza et al. have demonstrated that the silencing of *HMOX1* in FH-deficient cell lines resulted in their synthetic lethality [16]. This term refers to the situation in which simultaneous defect in two genes results in cell death, whereas at the same time separate dysfunction or mutation of each gene does not affect cell viability [17].

Several approaches for HO-1 silencing are used in experimental settings, with the main focus on RNA interference (RNAi) and pharmacologic inhibition of HO-1 activity. Despite numerous advantages of both strategies, their therapeutic application, at least in some cases, are strongly limited. One of the drawbacks of the RNAi approach might be related to its off-target effects causing inhibition of other undesired genes. The comprehensive computational study emphasized these off-target effects which may lead to misinterpretation of obtained results [18]. On the other hand, the disadvantages of commercially available metalloporphyrin-based inhibitors of heme oxygenase activity, such as inducing *HMOX1* expression and the lack of selectivity towards HO isoforms, are well-known [15,19]. It is especially important as HO-2 isoform, on the contrary to the inducible HO-1 is constitutively expressed and is responsible for the maintenance of cellular homeostasis and for example the viability of endothelial cells [20,21,22]. To overcome these problems we [23] and others [24] have recently elaborated on the new, imidazole-based compounds exerting some properties superior to known inhibitors of HO activity.

In the current study, we hypothesized that HO-1 inhibition will result in an anti-cancer effect in the examined FH-deficient cell lines. Specifically, we aimed at the verification of the synthetic lethality concept of *HMOX1* and *FH* in three different HLRCC cell lines using not only genetic but also pharmacological inhibition of *HMOX1*. We were especially interested in checking the effectiveness of SLV-11999 inhibitor, described by us recently as the anti-cancer compound in pancreatic and prostate cancer cell lines [23]. We have demonstrated that both strategies hamper cancer cell viability and self-renewal capacity and confirmed that the concept of synthetically lethal interactions of *HMOX1* and *FH* genes might be an attractive option for the treatment of HLRCC-associated tumors.

## 2. Materials and Methods

### 2.1. Cell Culture

Human FH-deficient cancer cell lines (UOK 262, UOK 268, and NCCFH1), as well as human kidney cell line lines HK-2 (ATCC CRL-2190) and HEK293 (kindly obtained from dr Maciej Wiznerowicz from Wielkopolskie Centrum Onkologii, Poznan, Poland), were cultured in DMEM HG (Lonza, Basel, Switzerland), supplemented with 10% FBS (Biowest/EURx) and antibiotics, 100 U/mL penicillin, and 100 μg/mL streptomycin (Lonza, Basel, Switzerland). UOK 262 and UOK 268 cell lines were kindly provided by Prof. W. M. Linehan (Center for Cancer Research, National Cancer Institute, Bethesda, MD, USA) [16], whereas NCCFH1 were donated by Prof. Min-Han Tan (Department of Biodevices and Diagnostics, Institute of Bioengineering and Nanotechnology, Singapore, Republic of Singapore) [17].

Cells were maintained under standard conditions (37 °C, 5% CO_2_, 95% humidity). Mycoplasma contamination was checked routinely in all cell lines using MycoAlert Mycoplasma Detection Kit (Lonza, Basel, Switzerland), following manufacturer’s instructions.

### 2.2. Drugs and Reagents

For pharmacological inhibitions various compounds were tested. SLV-11199 (IC_50_ = 2.23 ± 0.35 µM for HO-1 and IC_50_ = 1.07 µM for HO-2) [23] and QC-308 (IC_50_ = 1.25 ± 0.049 μM for HO-1 and IC50 = 1.12 ± 0.028 µM for HO-2, unpublished data) were synthesized as previously described [23]. SnPPIX (IC50 = 0.47 µM for HO-1 and IC50 = 0.18 µM for HO-2), and ZnPPIX (IC50 = 5.45 µM for HO-1 and IC_50_ = 2.65 µM for HO-2) [25] were purchased from Frontier Scientific (Logan UT, USA).

### 2.3. Production of Lentiviral Vectors Encoding shRNA Sequences against *HMOX1* and Transduction of Target Cells

shRNA constructs (shHO-1) against human *HMOX1* and non-targeting shRNA (scrambled shRNA) in pGFP-C-shLenti vectors were purchased from OriGene (Rockville, MD, USA) and were used for the production of lentiviral vectors in HEK293 cells according to the protocol described elsewhere [19]. After lentiviral vectors titration, 20 MOI (multiplicity of infection) of vectors encoding shRNA sequences against *HMOX1* and scrambled shRNA were used. The transduction process was facilitated by the addition of 5 µg/mL polybrene to the infection solution. Transduction efficiency was monitored under the fluorescent microscope by the assessment of green fluorescent protein (GFP) positive cells.

### 2.4. MTT (3-(4,5-Dimethylthiazol-2-yl)-2,5-Diphenyltetrazolium Bromide) Viability Assay

To determine the viability of cancer cells, MTT (3-(4,5-dimethylthiazol-2-yl)-2,5-diphenyltetrazolium bromide) assay was used (Sigma-Aldrich, St. Louis, MO, USA). After cell seeding and adequate treatment (depending on the experiment, specified in (Results section) medium was removed, cells were carefully washed with PBS and stained with 1 mg/mL MTT in the medium for 2 h at 37 °C. After that, formazan crystals were dissolved in 100 µL/well of lysis buffer consisting of 0.6% (*v*/*v*) acetic acid (Avantor Performance Materials Poland, Gliwice, Poland) and 10% (*w*/*v*) SDS (BioShop, Burlington, ON, Canada) in DMSO (Chempur, Piekary Śląskie, Poland). Absorbance at 570 nm (reference wavelength: 690 nm) was measured using a Tecan Infinite M200 microplate reader. Mean signal from blank control wells containing non-stimulated cells that had not been stained with MTT, was subtracted from all readings.

### 2.5. BrdU (Bromodeoxyuridine) Incorporation Assay

To assess the proliferation of cancer cells after genetic inhibition of *HMOX1*, BrdU (bromodeoxyuridine) incorporation colorimetric assay was performed (Roche, Basel, Switzerland) following the manufacturer’s instructions.

### 2.6. Colony Formation Assay

Colony formation assay was performed in order to evaluate the impact of chemical HO activity inhibitors on the ability of cancer cells to form colonies. A total of 1500 cells were seeded in 12-well plates and grown for 10 days for the experiments with HO-1 inhibitors and for 14 days for shRNA. Afterward, cells were fixed with cold (–20 °C), 100% methanol for 20 min on ice and stained with 0.05% (*w*/*v*) crystal violet (BioShop, Burlington, ON, Canada) in 20% methanol for 20 min at room temperature. Crystal violet was then precisely removed and pictures of the plates were taken using Fusion FX5 XT camera (Vilber, Collégien, France).

### 2.7. Quantitative Real-Time PCR (qRT-PCR)

RNA was isolated according to Chomczynski and Sacchi [26] using Fenozol (A&A Biotechnology, Gdynia, Poland). Reverse transcription was performed with RevertAid Reverse Transcriptase polymerase (Thermo Fisher Scientific, Waltham, MA, USA) after confirmation of the RNA concentration and purity on a NanoDrop 1000 (Thermo Fisher Scientific, Waltham, MA, USA)F. qRT-PCR with SybrGreen Mix (Sigma-Aldrich, St. Louis, MO, USA) was performed using specific primers (Table 1). Eukaryotic translation elongation factor 2 (*EEF2*) was used for gene expression normalization. The reaction was performed using a StepOnePlus TM Real-time PCR System (Applied Biosystems, Foster City, CA, USA). The relative gene expression level was calculated as 2^−ΔC^_T_, where ΔC_T_ is defined as a difference between C_T_ values obtained for the gene of interest and housekeeping gene *EEF-2*. Data were normalized to the control cells.

### 2.8. Determination of Protein Concentration

Protein concentration was determined by BCA (bicinchoninic acid) assay (Sigma-Aldrich, St. Louis, MO, USA). Briefly, 3 µL of protein lysates and BSA standards (bovine serum albumin, concentration range of 0–10 mg/mL, BioShop, Burlington, ON, Canada;) were transferred into 96-well plate in duplicate. Then, 100 µL of reaction mixture consisting of BCA and CuSO_4_ (50:1 ratio) was added into each well. The plate was incubated at 37 °C in the dark until sufficient color had developed, then the absorbance at 562 nm using Tecan Infinite M200 (Männedorf, Switzerland) microplate reader was measured.

### 2.9. Fumarate Hydratase Activity Measurement

In order to check FH activity in FH-deficient and control cell lines, Fumarase Activity Colorimetric Assay was performed according to the manufacturer’s instructions (BioVision, Milpitas, CA, USA) using Tecan Infinite M200 microplate reader. Results were normalized to the protein content of each sample determined by the BCA method. FH activity was calculated as ng of nicotinamide adenine dinucleotide (NADH) produced in 1 min by 1 mg of protein at 37 °C.

### 2.10. Western Blot

To determine HO-1, NAD(P)H quinone dehydrogenase 1 (NQO1) and FH expression on the protein level in analyzed cell lines and after transduction, cultured cells were lysed with RIPA buffer (Sigma-Aldrich, St. Louis, MO, USA) and 25–30 µg of protein lysates were loaded on 12% SDS-PAGE gel followed by electrophoresis and overnight wet transfer of proteins to nitrocellulose membrane at 30 V. Membranes were blocked with 5% non-fat milk in TBS (Tris-buffered saline, BioShop, Burlington, ON, Canada) + 0.1% (*v*/*v*) Tween-20 (BioShop, Burlington, ON, Canada) for 1.5 h at room temperature. Primary antibodies diluted in blocking solution: Rabbit anti-HO-1 (ADI-SPA-894, Enzo Life Science, Farmingdale, NY, USA, 1:500), mouse anti-α-tubulin (CP06, Calbiochem, San Diego, CA, USA, 1:1000), mouse anti-FH (ab-58232, Abcam, 1:500), and rabbit anti-NQO1 (ab-34173, Abcam, 1:500) were used for overnight incubation at 4 °C. The next day, membranes were washed and incubated with secondary antibodies conjugated with HRP: Goat anti-mouse (BD Pharmingen, San Diego, CA, USA, 1:5000 for FH, 1:10000 for tubulin) and goat anti-rabbit (Cell Signaling Technology, Danvers, MA, USA, 1:5000) for 1 h at room temperature. After a series of washings, a luminescent substrate for HRP activity (Immobilon Chemiluminescent HRP Substrate, Merck Millipore, Billerica, MA, USA) was added for 10 min and membranes were manually developed on X-ray film.

### 2.11. Oxygen Consumption Rate (OCR) and Extracellular Acidification Rate (ECAR) Measurement

Oxygen consumption rate (OCR) and extracellular acidification rate (ECAR) in HK-2, UOK 262, UOK 268, and NCCFH1 cells were measured using Seahorse Bioscience XFe96 Analyzer (Agilent Technologies, Santa Clara, CA, USA). After optimization 20,000 cells were seeded into Seahorse XFe96-well plates 24 h before the experiment. On the day of the experiment medium was changed for low-buffered assay medium containing DMEM base (8.3 g/L, Sigma-Aldrich, St. Louis, MO, USA), L-Glutamine 2 mM (Sigma-Aldrich), and 0.5% phenol red (Sigma-Aldrich, St. Louis, MO, USA ) (pH: 7.4) and incubated at 37 °C, 20% O_2_, without CO_2_ for about 1 h. Changes in glycolysis (based on ECAR values) were assessed in an XF glycolysis stress test where sequential injections of 10 mM glucose, 1 μg/mL oligomycin, and 10 mM 2-DG were performed. OCR values served as an indicator of the oxidative phosphorylation. Based on ECAR values glycolysis (maximal rate measurement after glucose injection minus measurement prior to glucose injection), glycolytic capacity (maximal rate measurement after oligomycin injection minus measurement prior to glucose injection), and glycolytic reserve (glycolytic capacity minus glycolysis) were calculated.

### 2.12. Statistical Analysis

All experiments were performed in duplicate or triplicate and were repeated two or three times. Results are presented as mean ± SD. Statistical analysis was performed in GraphPad Prism 8 Software using Student’s *t*-test or ANOVA test. Results were considered statistically significant at *p* < 0.05.

## 3. Results

### 3.1. Characterization of FH-Deficient Cell Lines

So far, only three immortalized human cell lines derived from patients suffering from HLRCC disease were established and described: UOK 262 cell line isolated from retroperitoneal lymph node metastasis [4], UOK 268 cell line isolated from primary kidney cancer [27] and, most recently, NCCFH1 cell line isolated from lung metastasis [28]. Initially, we aimed at the direct comparison of all cell lines with non-malignant human kidney cell line, HK-2, in terms of FH expression and other cell characteristics.

**Figure 1:**
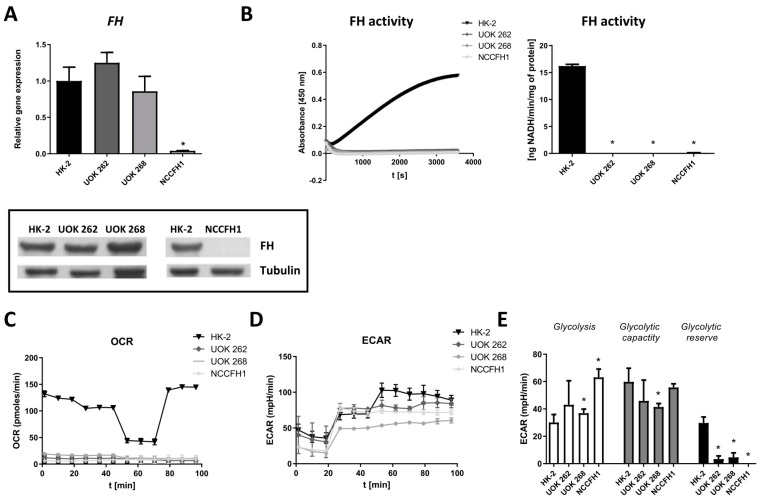
Characterization of fumarate hydratase (FH)-deficient cell lines. (**A**) qRT-PCR results (upper panel) and Western blot results (lower panel) for *FH* mRNA and protein level in UOK 262, UOK 268 and NCCFH1 cell lines in comparison to HK-2 cell lines. qRT-PCR results presented as relative fold change (mean ± SD). (**B**) FH activity in UOK 262, UOK 268, NCCFH1 and HK-2 cell lines, presented as absorbance changes over time (left panel) and ng NADH produced per minute by 1 mg protein (right panel) (mean ± SD). (**C**) Oxygen consumption rate (OCR) presented as a pmol/min and (**D**) extracellular acidification rate (ECAR) in UOK 262, UOK 268, NCCFH1, and HK-2 cell lines presented as an mpH/min. (**E**) Calculation of the typical glycolytic parameters based on Seahorse analysis: Glycolysis, glycolytic capacity, and glycolytic reserve shown as mpH/min (mean ± SD). * *p* < 0.05 vs. HK-2 cell line, Student’s *t*-test.

*FH* mRNA and protein level (Figure 1A) were detectable in UOK 262 and UOK 268 cell lines, whereas, in NCCFH1 cells, no signal could be detected using both qRT-PCR and immunoblot analysis (Figure 1A). Of importance, all FH-deficient cell lines lacked the enzymatic activity of FH (Figure 1B).

In comparison to normal kidney cells, all FH-deficient cell lines exhibited attenuated mitochondrial respiration (Figure 1C) with the preferential utilization of the glycolysis to produce ATP in normoxic conditions (Figure 1D,E). Moreover, all three cell lines seem to exploit glycolysis at the maximal rate as very low (UOK 262 and UOK 268) or even no (NCCFH1) glycolytic reserves could be estimated when Glycolysis Stress Test was performed (Figure 1E).

**Figure 2:**
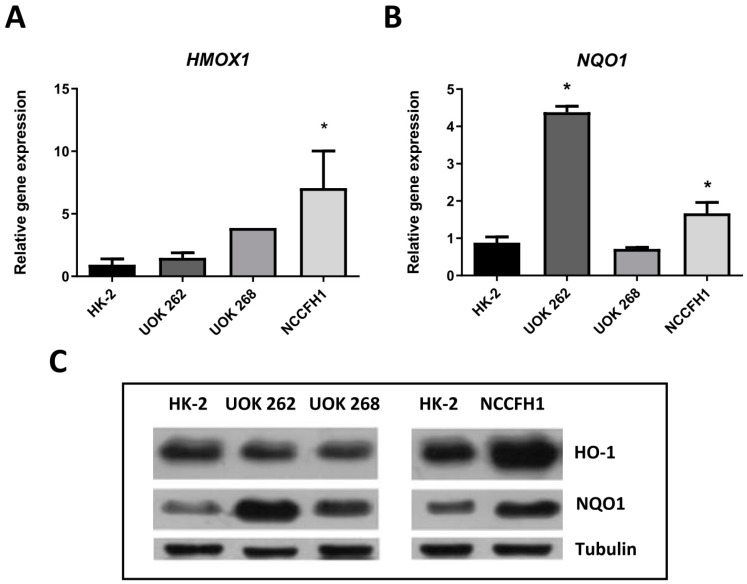
Analysis of *HMOX1* and *NQO1* mRNA and protein level in FH-deficient cell lines. qRT-PCR results of heme oxygenase-1 (*HMOX1*) (**A**) and NAD(P)H quinone dehydrogenase 1 (*NQO1*) (**B**) expression presented as relative fold change (mean ± SD). (**C**) Representative picture of Western blot analysis of heme oxygenase-1 (HO-1) and NQO1 protein levels. * *p* < 0.05 vs. HK-2 cell line, Student’s *t*-test.

Further analysis revealed an increase in the expression of Nrf2-regulated genes (Figure 2A,B). *HMOX1* mRNA level was upregulated in UOK 268 and NCCFH1 cell lines in comparison to the control cell line (Figure 2A). Minor differences in *NQO1* were observed between NCCFH1 and HK-2 cell lines, whereas a potent increase was visible in UOK 262 (Figure 2B). On the protein level, HO-1 upregulation was observed in the NCCFH1 cell line, with no apparent changes in both UOK cell lines (Figure 2C). NQO1 protein level was elevated in all FH-deficient cell lines with the strongest changes being observed in UOK 262 (Figure 2C).

### 3.2. Genetic Inhibition of *HMOX1* Decreases Viability and Proliferation of FH-Deficient Cell Lines

**Figure 3:**
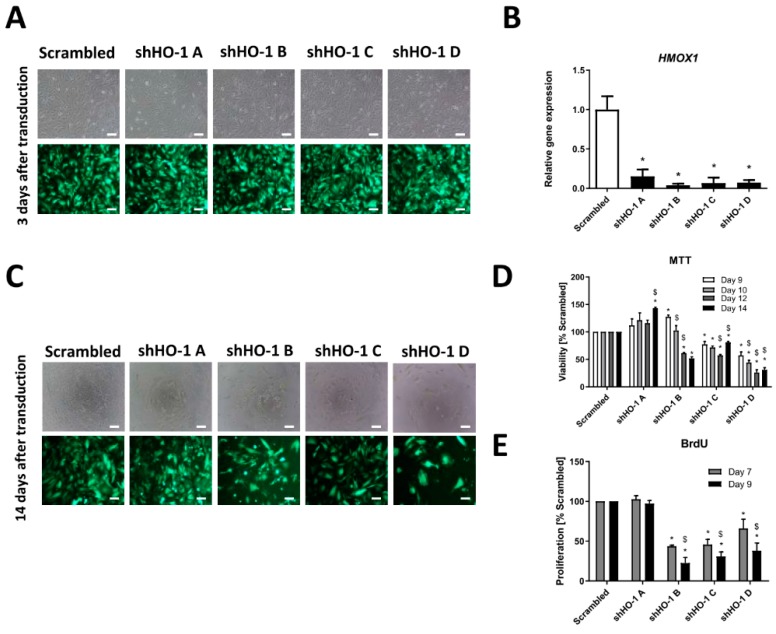
Genetic inhibition of *HMOX1* in the UOK 262 cell line leads to decreased cell viability after transduction with three out of four tested shRNA. UOK 262 cell line was transduced with lentiviral vectors encoding four shRNA sequences against *HMOX1* transcript and with one, non-specific scrambled shRNA. (**A**) Representative pictures of UOK 262 cells 3 days after transduction, scale bar: 100 µm, upper row—bright field, lower row—fluorescence. (**B**) qRT-PCR results of *HMOX1* silencing level in UOK 262 cell line after transduction with HO-1 shRNA sequences and scrambled shRNA presented as relative fold change (mean ± SD). (**C**) Microscopic images both in the bright field (upper part) and under fluorescence (lower part) 14 days after transduction with lentiviral vectors encoding shHO-1 and scrambled sequence showing decreased cell number in shHO-1 B-D. (**D**) MTT (3-(4,5-dimethylthiazol-2-yl)-2,5-diphenyltetrazolium bromide) viability assay after several time-points (9–14 days) from transduction, presented as the percentage of scrambled shRNA (mean ± SD). (**E**) BrdU (bromodeoxyuridine) incorporation assay performed 7 and 9 days after transduction, presented as the percentage of scrambled shRNA (mean ± SD). * *p* < 0.05 vs. scrambled sequence, Student’s *t*-test; $ *p* < 0.05 vs. previous tested day of the assay, ANOVA test.

Since *HMOX1* inhibition in FH-deficient cells was shown to be synthetically lethal [16], we aimed to validate the effect of genetic inhibition of *HMOX1* on viability and proliferation of UOK 262 cell line. In order to do this, cells were transduced with lentiviral vectors encoding four shRNA sequences specifically targeting *HMOX1* transcript and one non-*HMOX1* specific transcript with scrambled shRNA. Transduction efficiency was high as assessed by the presence of GFP positive cells (Figure 3A). Additionally, the level of HO-1 silencing in the case of all shRNA sequences was pronounced as determined by qRT-PCR (Figure 3B).

We monitored the viability of UOK 262 after several time-points (9–14 days) from transduction. We observed significantly decreased viability of cells transduced with three out of four used shRNA sequences in comparison to scrambled shRNA (Figure 3C,D).

Particularly after 14 days from transduction, the significant decrease in cell viability after treatment with shHO-1 B-D was observed by direct inspection under the microscope both in the bright field and under fluorescence modes (Figure 3C) as well as in the MTT assay (Figure 3D). The proliferation of UOK 262 cells was assessed by BrdU proliferation test and a pronounced decrease in cell proliferation transduced with shRNA sequences B-D was also noted 7 and 9 days after transduction (Figure 3E). Interestingly, transduction with shRNA sequence A did not impair UOK 262 cell line viability and proliferation at any analyzed time-point after transduction (Figure 3C–E), although inhibition of HO-1 expression was prominent (Figure 3B). However, the rate of inhibition was lower than in the case of other sequences.

**Figure 4:**
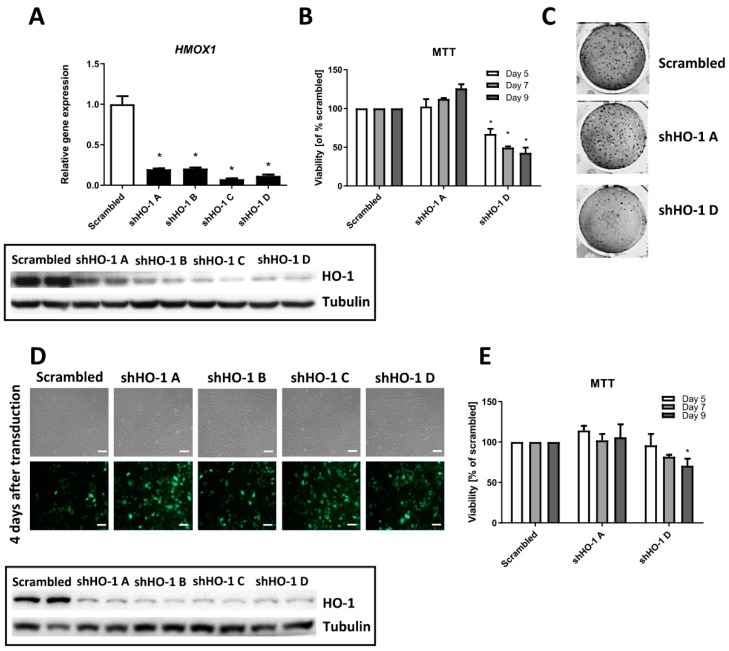
Genetic inhibition of *HMOX1* in the NCCFH1 and UOK 268 cell lines exert similar effects as in UOK 262 cells. FH-deficient cell lines were transduced with lentiviral vectors encoding four shRNA sequences against *HMOX1* transcript and with one, non-specific scrambled shRNA. (**A**) qRT-PCR results of *HMOX1* silencing level in NCCFH1 cell line after transduction with HO-1 shRNA sequences and scrambled shRNA presented as relative fold change (mean ± SD, upper panel); a representative picture of Western blot analysis of HO-1 after transduction (lower panel). (**B**) MTT viability assay on NCCFH1 cells after several time-points (5–9 days) from transduction, presented as the percentage of scrambled shRNA (mean ± SD). (**C**) Representative pictures of colony formation assay on the NCCFH1 cell line after 14 days after transduction with shHO-1. (**D**) Microscopic images of UOK268 cells both in the bright field (upper part) and under fluorescence (lower part) 4 days after transduction with lentiviral vectors encoding shHO-1 and scrambled; scale bar: 100 µm. (**E**) MTT viability assay on UOK268 cells after several time-points (5–9 days) from transduction, presented as the percentage of scrambled shRNA (mean ± SD). * *p* < 0.05 vs. scrambled sequence, Student’s *t*-test.

Noteworthy, we observed similar effects in the NCCFH1 (Figure 4A–C) cell line. All tested sequences led to the inhibition of the HO-1 level (Figure 4A) but the differential effect on cell viability and self-renewal capacity was observed when we compared the effectiveness of sequence A and sequence D.

Accordingly to the effect in UOK 262 cell line, transduction with shHO-1 A did not exert any effect whereas the shHO-1 D sequence resulted in approximately 50% inhibition of cell viability 5, 7, and 9 days after transduction (Figure 4B). The clonogenic potential was affected in a similar way (Figure 4C). In the last tested cell line, UOK 268, despite the high transduction efficiency and the pronounced rate of HO-1 inhibition (Figure 4D), the viability was only slightly reduced in cells transduced with shHO-1 D sequence (Figure 4E). Again, although *HMOX1* inhibition after sequence A was visible (Figure 4D), no effect in MTT assay was observed (Figure 4E).

### 3.3. The Effect of Chemical Inhibition of HO Activity on Viability and Clonogenic Potential of FH-Deficient Cell Lines

**Figure 5:**
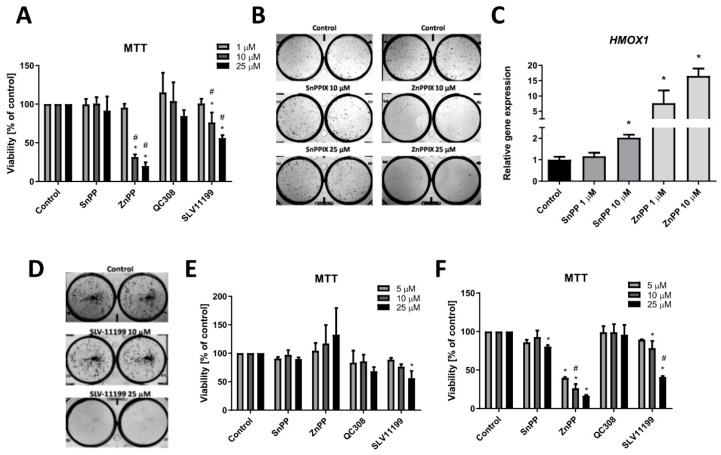
The effect of HO activity inhibitors on FH-deficient cell viability and clonogenic potential. (**A**) Treatment of UOK 262 cell line with 1–25 µM SnPPIX/ZnPPIX for 72 h. MTT assay results presented as the percentage of control, non-stimulated cells (mean ± SD). (**B**) Representative pictures of colony formation assay on the UOK 262 cell line after 10 days of stimulation with 10 and 25 µM SnPPIX/ZnPPIX. (**C**) qRT-PCR results of *HMOX1* expression in UOK 262 cells after 24 h stimulation with 1 and 10 µM SnPPIX/ZnPPIX. Results presented as relative fold change (mean ± SD) (**D**) Representative pictures of colony formation assay on the UOK 262 cell line after 10 days of stimulation with SLV-11199. NCCFH1 (**E**) and UOK 268 (**F**) cell viability after treatment with SnPPIX, ZnPPIX, QC-308, and SLV-11199 for 72 h. MTT assay results presented as percentage of control, not stimulated cells (mean ± SD). * *p* < 0.05 vs. control cells, Student’s *t*-test; # *p* < 0.05, vs. lower concentration, ANOVA test.

Having found the differential response of the cells transduced with various shRNA sequences indicating the possible off-target effect, we investigated the pharmacological inhibition of HO activity on UOK 262 cells viability and clonogenic potential. Commercially available inhibitors, tin and zinc protoporphyrin (SnPPIX and ZnPPIX, respectively) were used at first. UOK 262 cell line was treated with 1–25 µM SnPPIX and ZnPPIX for 72 h and subsequently, MTT test was performed. As it is shown in Figure 5A, no effect was observed after stimulation with SnPPIX. On the contrary, treatment with higher concentrations of ZnPPIX resulted in a significant decrease in UOK 262 cell viability.

Clonogenic potential (Figure 5B) of UOK 262 cells upon after stimulation with 10 and 25 µM SnPPIX was not affected; however, ZnPPIX diminished the colony formation potential at 10 µM concentration. We and others previously demonstrated that metalloporphyrins may significantly induce *HMOX1* mRNA level [15,19], thus, we evaluated if such undesired effects are present in our models. In UOK 262 cells, both ZnPPIX and 10 µM SnPPIX demonstrated the stimulatory effect on *HMOX1* expression (Figure 5C).

A similar examination was performed for the novel, small-molecule inhibitor of HO activity described recently by us [29]. As a reference, non-porphyrin HO activity inhibitor QC-308 was used [30]. UOK 262 cells were treated with 1, 10, and 25 µM SLV-11199 and QC-308 and cell viability was assessed. We observed not only decreased viability upon SLV-11199 treatment (Figure 5A) but also attenuated clonogenic potential in the case of 25 µM concentration (Figure 5D). No differences in cell viability were observed for QC-308 (Figure 5D). Interestingly, ZnPPIX was not effective in NCCFH1 (Figure 5E) whereas it was working potently in UOK 268 (Figure 5F). However, similar effects in cell viability were noted when NCCFH1 (Figure 5E) and UOK 268 (Figure 5F) were treated with SLV-11199, confirming high potency of this new inhibitor.

## 4. Discussion

HLRCC syndrome, characterized by the loss-of-function mutation in the *FH* gene, is associated with aggressive kidney cancers, prone to metastasize early [2]. The major finding of the present study is the confirmation that indeed inhibition of *HMOX1* and *FH* genes results in synthetic lethality. Moreover, we showed the potency of new HO activity inhibitor, SLV-11199, and its possible anti-cancer application in FH-deficient cell lines. The silent finding of our study underlines that the observed effect might be dependent on the cell type, shRNA sequence, and HO-1 inhibitor used.

The characterization of cell lines derived from patients suffering from HLRCC disease revealed typical features of HLRCC pathology. Both *FH* mRNA and protein levels were detectable in UOK 262 and UOK 268 cell lines, but in comparison to non-malignant human kidney cell line, they lacked FH enzymatic activity. In the NCCFH1 cell line, even *FH* mRNA and protein were both on the undetectable level. Obtained results are in agreement with those published by Yang et al. [4] and Perrier-Trudova et al. [28] emphasizing loss-of-function defect in the *FH* gene.

Moreover, we observed strong glycolytic bias in all three cell lines. Studies of both ECAR (showing the level of glycolysis) and OCR (corresponding to the level of oxygen respiration), confirmed that in UOK 262, UOK 268, and NCCFH1 cell lines the basic level of glycolysis is increased in comparison to control HK-2 cells. These results, combined with the almost complete absence of ATP production in the mitochondrial electron transport chain, allow presuming that FH deficiency leads to the Warburg effect. Our observation confirms the literature data, showing that in cells isolated from patients with HLRCC [28] as well as in murine cells lacking FH [16] significant increase in glycolysis and negligible oxygen consumption were also evidenced. Moreover, to our best knowledge, we reported for the first time that the glycolytic reserve in the tested cell lines is minimal, suggesting that FH-deficient cells utilize glycolysis at the maximal glycolytic capacity. As our and other data indicate, FH-deficient cells rely mainly on glycolysis. Despite that, they still utilize glutamine, which replaces glucose as a main source of carbons, in mitochondrial reductive carboxylation for partial production of NADH [31]. In FH-deficient cells, glucose is shunted into the glycolysis and pentose phosphate pathway [32].

Further analysis showed upregulation of *HMOX1* and *NQO1* in examined cell lines in comparison to normal kidney cells. This is in agreement with the analysis done by Adam et al., that pointed out the relevance of the Nrf2-related anti-oxidant pathway in the development of HLRCC-associated kidney cancer [13]. Among others, its involvement in heme metabolism seems to play a crucial role. Interestingly, in silico study that was subsequently confirmed experimentally, pointed out the importance of heme biosynthesis and degradation pathways in FH-deficient cells linking glutamine uptake (as a source of carbon required for heme biosynthesis) with bilirubin excretion. Indeed, 18 out of 24 genes predicted as synthetically lethal with *FH* were associated with heme metabolism pathways. Noteworthy, high throughput screening performed on FH-deficient cells revealed that also multiple glycolysis-related genes and adenylate cyclases are essential for the survival of these cells [29].

We have previously shown that HO-1 is essential for proliferation, angiogenesis, and metastasis of different tumor types, including melanoma, rhabdomyosarcoma, and pancreatic cancer [23,33,34]. Of importance, in the study done by Frezza et al., inhibition of *HMOX1* was reported to be synthetically lethal for FH-deficient mouse kidney cells [16]. As we are aware of many limitations of both genetic and chemical inhibition of *HMOX1* and possible cell-type-dependent effects, we decided to investigate the effect of two strategies for inhibition of HO-1 in various FH-deficient cell lines (to check if the effect is not restricted to one cell line but rather represents a general phenomenon). As demonstrated in this study, genetic inhibition of *HMOX1* significantly diminished both the viability and proliferation of UOK 262 as evidenced after several time-points after transduction with lentiviral vectors encoding shRNAs against *HMOX1*. This was accompanied by the morphological abnormalities of the UOK 262 cell line. However, whether observed alterations are driven by apoptosis, autophagy, or changes in the cell cycle regulation, remains to be established.

Importantly, we observed some phenotypic differences in the UOK 262 cell line transduced with one shHO-1 sequence compared to three other shHO-1 sequences. Although the level of *HMOX1* inhibition was quite prominent, neither viability nor proliferation of the cells transduced with shHO-1 sequence A was impaired. A similar situation was also demonstrated in two other FH-deficient cell lines, as we again observed no effect on cell viability of the same shRNA sequence despite the potent inhibitory effect on *HMOX1* mRNA and protein levels. This could be explained by the off-target effect of this particular shRNA sequence that, except silencing of *HMOX1*, could also inhibit some other genes. Such effect is generally considered as a disadvantage of the shRNA approach [18] and it underlines the importance of using several shRNA sequences and not to restrict to only one. Another alternative for future experiments could be the CRISPR/Cas9-mediated gene silencing approach, which, in comparison to shRNA, is known to be less susceptible to off-target effects than RNA interference [35] and may give better results in terms of inhibition of HO activity [19]. Although we did not investigate this very carefully, we cannot exclude the possibility that a certain extent of HO-1 inhibition is also required to cause decreased cell viability. Nevertheless, it seems unlikely in our case, as we obtained very potent inhibition for all tested shRNAs with the various phenotypic outcome for one shRNA sequence. Furthermore, in line with the results obtained for FH-deficient cells, our unpublished results performed on different cancerous cell lines also confirmed that shHO-1 A, although it leads to the potent inhibition of HO-1, does not affect cell viability, unlike three other tested shRNA sequences. Thus, as all RNA interference-based approaches are known to have off-target effects resulting in off-target phenotypes [36,37] we speculated that this mechanism is responsible for divergent properties of one out of four tested shRNA sequences leading to the effect that is not related to the inhibition of the gene of interest.

The obtained results with genetic inhibition of *HMOX1* are generally in agreement with the concept of synthetical lethality of *HMOX1* and *FH* genes, postulated and proven by Frezza et al. [16]. This suggests the possible role of HO-1 as a novel target in HLRCC-related kidney cancer. However, due to the several limitations of the therapeutic application of RNAi-mediated gene silencing, pharmacological inhibition of HO activity seems to be a better alternative. To our best knowledge, only one study done by already mentioned Frezza et al. aimed at investigation of the effect of a known inhibitor of HO activity, ZnPPIX, on the clonogenic potential of *FH*-deficient mouse kidney cells and UOK 262 cell line. In both cases, treatment with ZnPPIX significantly diminished the clonogenic potential of *FH*-deficient cells, without affecting wild-type cells or cells with restored expression of correct *FH* gene. In our hands, a significant decrease in both viability and clonogenic potential of UOK 262 cells after stimulation with ZnPPIX was observed. Nonetheless, at the same time, ZnPPIX significantly upregulated HO-1 mRNA level. This is consistent with previously published reports indicating this stimulatory effect on *HMOX1* expression what may be, at least in some conditions, a serious drawback [15,19,23]. However, we evaluated the effect of ZnPPIX on the viability of other FH-deficient cell lines and in UOK 268 cells the results were comparable to UOK 262. Intriguingly, even the high concentration of this inhibitor did not affect NCCFH1 viability. Whether it is the effect of disturbed metalloporphyrin uptake or transport remains unknown. Interestingly, a new small-molecule inhibitor of HO activity, recently developed by us [23], was able to decrease cell viability and self-renewal capacity of UOK262 cells. Additionally, both UOK 268 and NCCFH1 treated with the highest concentration of SLV-11199 exerted diminished cell viability, although some subtle differences between those cell lines were observed. Of note, we have previously shown that SLV-11199 does not induce *HMOX1* expression, in other cancer cell lines, such as pancreatic PANC-1 or prostate DU-145 cells [23]. Nonetheless, further studies are warranted to expand those findings in FH-deficient cell lines, together with the evaluation of the possible mechanisms of cell death caused by the lack of HO-1 and FH.

## 5. Conclusions

To sum up, we demonstrated that inhibition of HO-1 can be considered as a potential anti-cancer strategy in HLRCC-related kidney cancer. Genetic inhibition of *HMOX1* by the shRNA approach, although shown to exert an anti-cancer effect, is still far from the therapeutic application. It might be also interesting to assess what is the effect of inhibition of the Nrf2 transcription factor, regulating *HMOX1* expression. On the other hand, pharmacological inhibition of HO activity resulted in diminished viability and clonogenic potential of cancer cells, but some limitations of known inhibitors and discrepancies obtained in the case of using SnPPIX and ZnPPIX have to be taken into consideration when planning both in vitro and in vivo experiments. A better alternative may provide novel, imidazole-based inhibitors of HO activity; however, more detailed studies are required in order to fully verify their potential as an anti-cancer agent.

## Figures and Tables

**Table 1:** Sequences of primers used in qRT-PCR.

Gene of Interest	Primer Sequence
*EEF2* forward	5′-GAGATCCAGTGTCCAGAGCAG-3′
*EEF2* reverse	5′-CTCGTTGACGGGCAGATAGG-3′
*FH* forward	5′-GTATTATGGCGCCCAGACC-3′
*FH* reverse	5′-ATCCTGGTTTACTTCAGCGG-3′
*HMOX1* forward	5′-TTCTTCACCTTCCCCAACATT-3′
*HMOX1* reverse	5′-CAGCTCCTGCAACTCCTCAAA-3′
*NQO1* forward	5′-AGGACCCTTCCGGAGTAAGA-3′
*NQO1* reverse	5′-CCAGGATTTGAATTCGGGCG-3′
